# Probability of COVID-19 Being the Culprit in Neurocognitive Deception: A Case Series of Incidental Strokes in ICU Patients With COVID-19

**DOI:** 10.7759/cureus.9857

**Published:** 2020-08-18

**Authors:** Ramakanth Pata, Roudabeh Kiani, Abolfazl Ahmady, Vanessa M Awad

**Affiliations:** 1 Pulmonary Medicine, Interfaith Medical Center, Brooklyn, USA; 2 Internal Medicine/Family Medicine, California Institute of Behavioral Neurosciences & Psychology, Fairfield, USA

**Keywords:** stroke, covid-19, sars-cov-2, anticoagulant, hemorrhage

## Abstract

The coronavirus disease 2019 (COVID-19) pandemic caused by the severe acute respiratory syndrome coronavirus 2 (SARS-CoV-2) virus, originated in Wuhan, China, and spread rapidly throughout the world, infecting millions and killing thousands. Although some patients have mild or even asymptomatic responses to this infection, hospitalized patients present with symptoms such as fever, cough, and difficulty breathing. Some patients have a severe response to the insult and experience rapid progression to acute respiratory distress and multiorgan failure. Furthermore, many patients developed complications due to this infection. Here, we present three patients who had strokes during their hospitalization for COVID-19 pneumonia.

## Introduction

In December 2019, coronavirus disease 2019 (COVID-19) quickly spread throughout the Wuhan province of China and around the world, which led to the World Health Organization (WHO) declaring it as a pandemic on March 11, 2020 [1]. COVID-19 is caused by the SARS-CoV-2 virus, a zoonotic coronavirus linked to bats similar to the severe acute respiratory syndrome (SARS) coronavirus and the Middle East respiratory syndrome (MERS) coronavirus [1].

SARS-CoV-2 is transmitted through bodily fluids, such as respiratory droplets, and survives longer at lower temperatures (i.e., 4°C has higher survival than 22°C) [1]. Additionally, it has a high incubation period (average 6.4 and range of 0-24 days) [2], reproductive number (R0 ranged from 1.4 to 6.49, with a mean of 3.28) [3], and reports have shown that the majority of patients are asymptomatic or have a mild response to the SARS-CoV-2 virus but release large amounts of viruses [2]. These factors together caused this virus to spread rapidly and kill hundreds of thousands of patients, which is more than the SARS pandemic in 2003 and the MERS pandemic in 2013 combined [2].

Patients with COVID-19 typically present with pyrexia, cough, and difficulty breathing. Furthermore, some patients experience constitutional symptoms such as myalgia, arthralgia, chills, and/ or gastrointestinal (GI) symptoms (i.e., nausea, vomiting, and diarrhea) [1]. In severe cases, patients experience severe insult to the lung tissue, causing acute respiratory distress syndrome. In addition to insults to the respiratory system, multiple complications in other systems have been reported in patients infected with COVID-19. These include, but are not limited to, acute kidney injury (AKI) [4] and cardiac injury [5]. This case series demonstrates three incidences of stroke in three patients hospitalized for COVID-19 pneumonia.

## Case presentation

Case 1

On April 11, 2020, a 33-year-old lady with a known medical history of diabetes mellites presented to the emergency department with altered mental status responsive to painful stimuli only. She was intubated due to a low Glasgow Coma Scale (GCS) during which she had 50-60 ml hematemesis (arterial blood gas results in Table 1). She was found to be tachycardic and tachypneic, with elevated white blood cell (WBC 29900/uL); subsequently, code sepsis was called. Other laboratory findings showed blood glucose 565 mg/dL, anion gap of 23, presence of ketones in urinalysis (UA), and creatinine 2.62 mg/dL. Other laboratory findings on admission are shown in Table 2.

**Table 1:** Case 1 Arterial blood gas from April 11, 2020, to April 22, 2020 Arterial blood gas: ABG; pCO2: partial pressure of carbon dioxide; pO2: partial pressure of oxygen; FiO2: fractional inspired oxygen; P/F: arterial oxygen partial pressure/fractional inspired oxygen

Date	4/11/2020	4/12/2020	4/13/2020	4/22/2020
PH	7.014	7.119	7.164	7.353
pCO2	49.7	34.8	31.7	38.8
pO2	62.5	383	290	129
O2 saturation	88.6%	99.6%	99.6%	98.6%
O2 delivery	Ventilator	Ventilator	Ventilator	Ventilator
FiO2	100%	100%	100%	50%
P/F ratio	62.5	383	290	258

**Table 2:** Laboratory results of all cases WBC: white blood cell; Hgb: hemoglobin; ESR: erythrocyte sedimentation rate; PT: prothrombin time; INR: international normalized ratio; APPT: activated partial thromboplastin time; CRP: C-reactive protein; AST: aspartate transaminase; ALT: alanine transaminase

	Case 1	Case 2	Case 3	Normal Range
WBC	29900/uL	8300/uL	6300/uL	4500-11000 /uL
Hgb	16.0 g/dL	18.4 g/dL	12.9 g/dL	M:13.5-17.5 g/dL F:12.0-15.5 g/dL
Platelet	426,000/uL	213000/uL	448,000/uL	150,000 to 450,000 /uL
ESR	76 mm/hr	83 mm/hr	67 mm/hr	0 – 22 mm/hr
PT	12 Sec	18.5 Sec	15.9 Sec	11 – 13.5 sec
INR	1.03	1.61	1.36	0.85-1.15
APTT	29.4 Sec	42.1 Sec	33.4 Sec	25-36 sec
D-dimer (max)	8077 (>18000) ng/ml	>18000 ng/ml	4491 (5796) ng/ml	0-500 ng/ml
Lactic acid	4.3 mmol/L	1.8 mmol/L	0.8 mmol/L	0.5-2.2 mmol/L
Lactate dehydrogenase	637 U/L	1186 U/L	905 U/L	140 – 280 U/L
Troponin I	0.00 ng/mL	0.14 ng/mL	0.00 ng/mL	< 0.03 ng/mL
Total creatinine kinase	152 U/L	1136 U/L	NA	22 – 198 U/L
CRP	47 mg/L	265 mg/L	NA	<10 mg/L
Ferritin	887 ng/mL	1514 ng/mL	5045 ng/mL	M:12-300 ng/mL F:12-150 ng/mL
Cr	2.62 mg/dL	1.90 mg/dL	1.23 mg/dL	0.84-1.21 mg/dL
Procalcitonin	0.35 ng/mL	NA	0.07 ng/mL	0.10-0.49 ng/mL
AST	NA	504 U/L (4/21/2020)	NA	5 to 40 U/L
ALT	NA	600 U/L (4/21/2020)	NA	7 to 56 U/L

**Figure 1:**
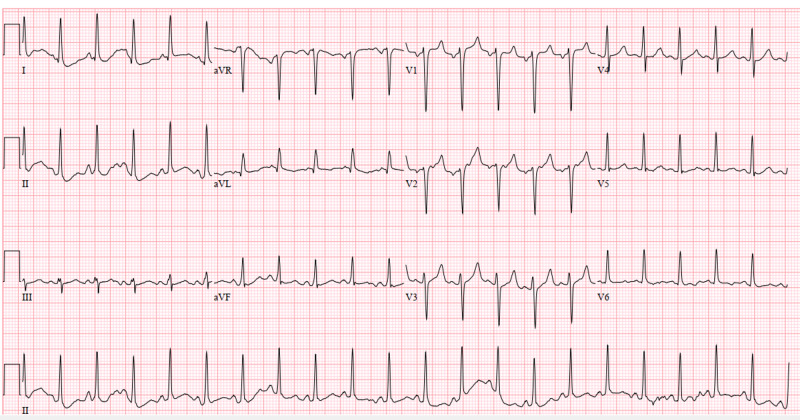
Case 1 electrocardiogram on admission (April 11, 2020)

**Figure 2:**
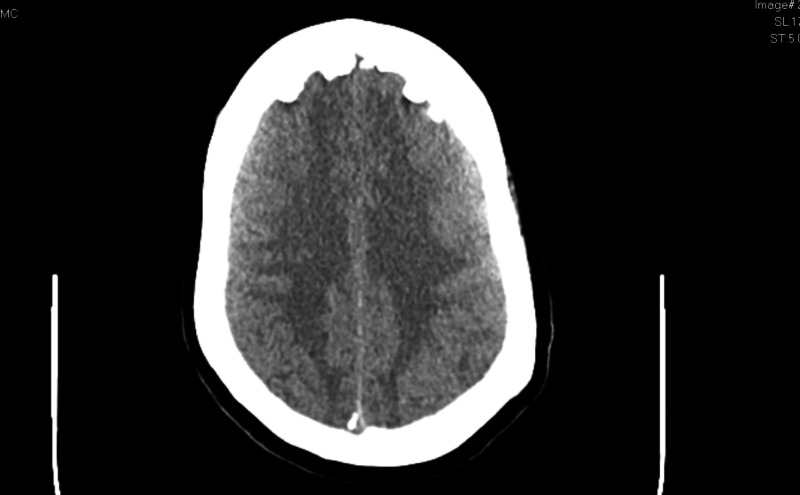
Case 1 head CT on admission April 11, 2020

**Figure 3:**
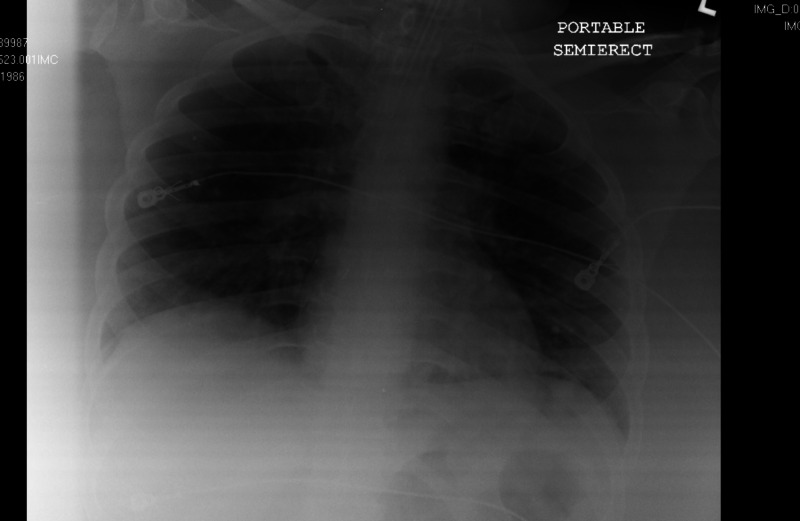
Case 1 chest X-ray on admission April 11, 2020

**Figure 4:**
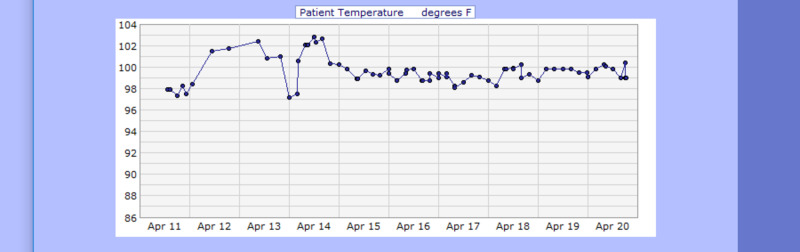
Case 1 temperature from April 11, 2020, to April 20, 2020 (F˚)

**Figure 5:**
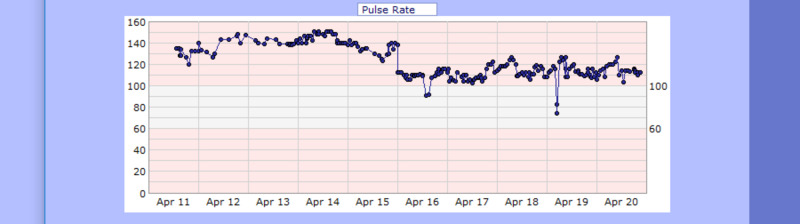
Case 1 pulse from April 11, 2020, to April 20, 2020 (bpm)

**Figure 6:**
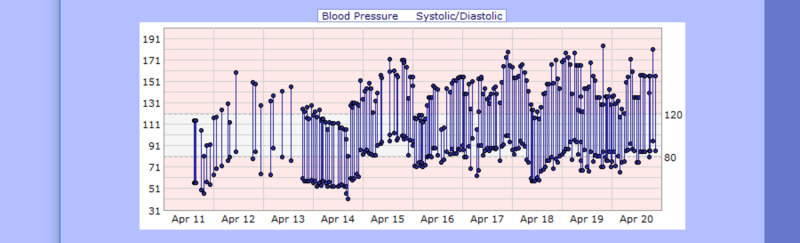
Case 1 blood pressure from April 11, 2020, to April 20, 2020 (mmHg)

**Figure 7:**
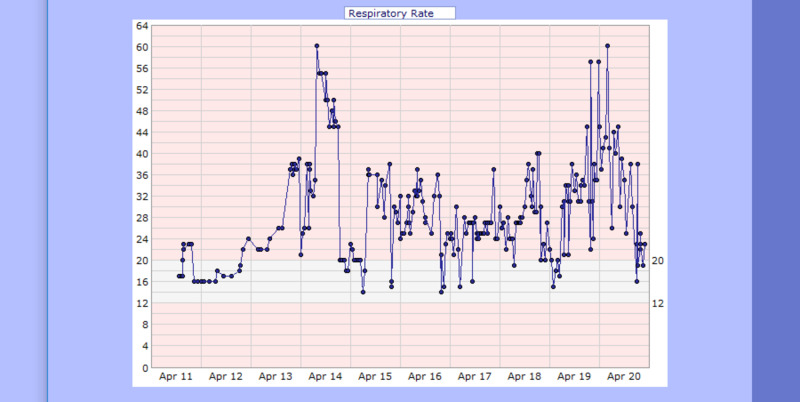
Case 1 respiratory rate from April 11, 2020, to April 20, 2020 (breaths per minute)

**Figure 8:**
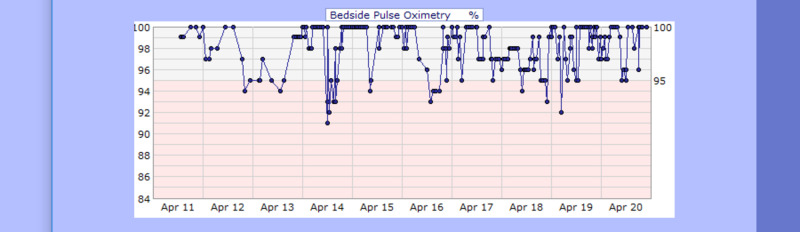
Case 1 pulse oximetry from April 11, 2020, to April 20, 2020 (%)

**Figure 9:**
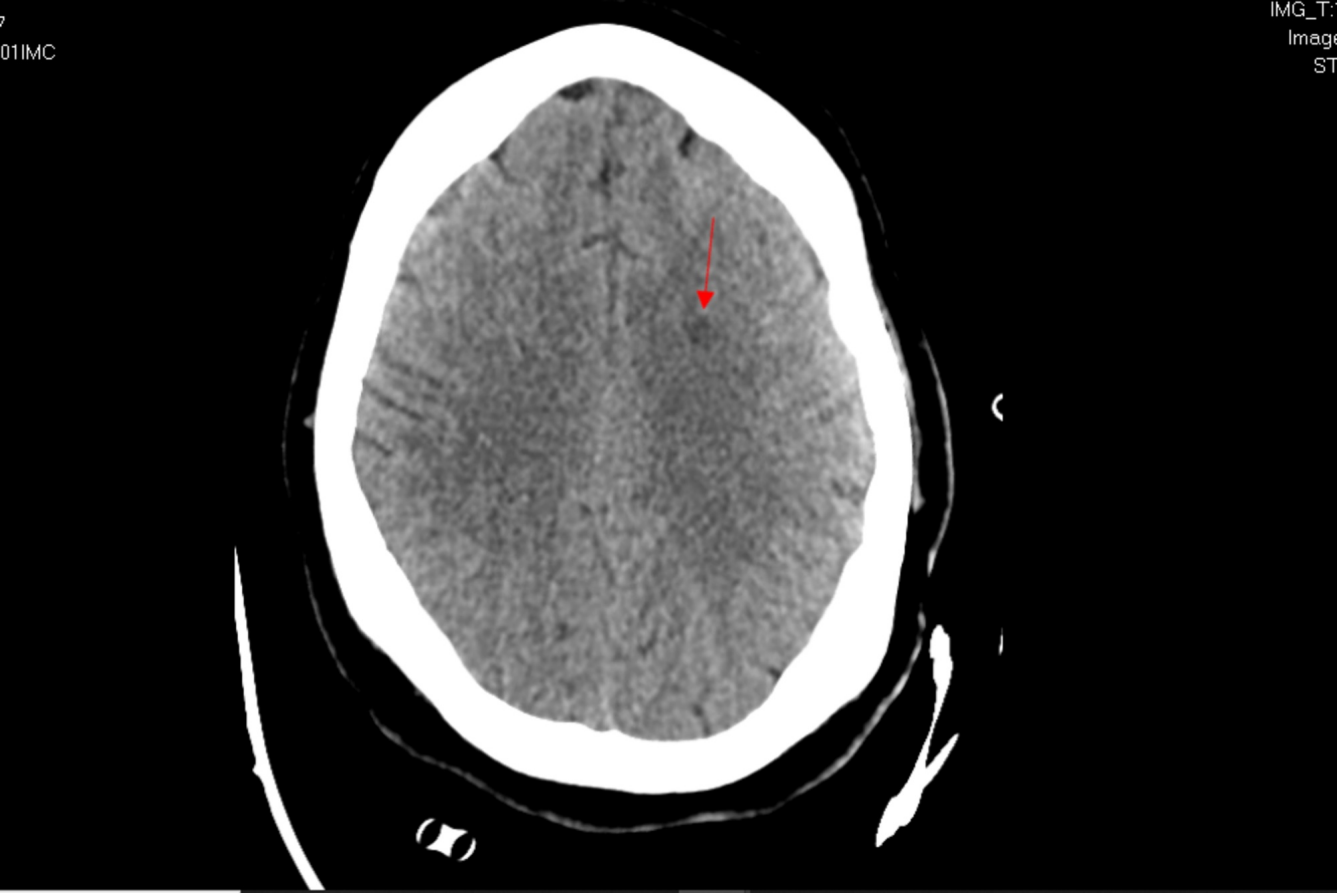
Case 1 repeat head CT on April 21, 2020

Additionally, the electrocardiogram showed sinus tachycardia with a corrected QT interval of 477 ms (Figure 1), and the head CT scan showed no acute intracranial abnormalities (Figure 2). Furthermore, a chest X-ray showed no acute pathologies (Figure 3), and the COVID-19 reverse transcriptase-polymerase chain reaction (RT-PCT) was performed due to the recent outbreak of the SARS-CoV-2 virus, which came back positive. She was admitted for diabetic ketoacidosis with hyperglycemic hyperosmolar syndrome, metabolic encephalopathy, acute kidney injury secondary to hypotension, COVID-19 pneumonia, and possible sepsis. She was given intravenous fluids, insulin drip, and antibiotics (vancomycin and meropenem) while on cardiac monitoring, and electrolyte levels were monitored (vitals presented in Figures 4-8). However, no anticoagulation was administered considering the hematemesis during intubation. On April 18, 2020, the fecal occult blood test was negative and hemoglobin levels remained stable; therefore, she was put on Lovenox® 100 mg subcutaneous (SQ) daily. A repeat of the head CT scan ordered on April 21, 2020, due to altered mentation and showed a trace amount of acute subarachnoid hemorrhage products along the left superior frontal sulcus and suspected acute lacunar infarction in the posterior left periventricular white matter (Figure 9).

Case 2

**Figure 10:**
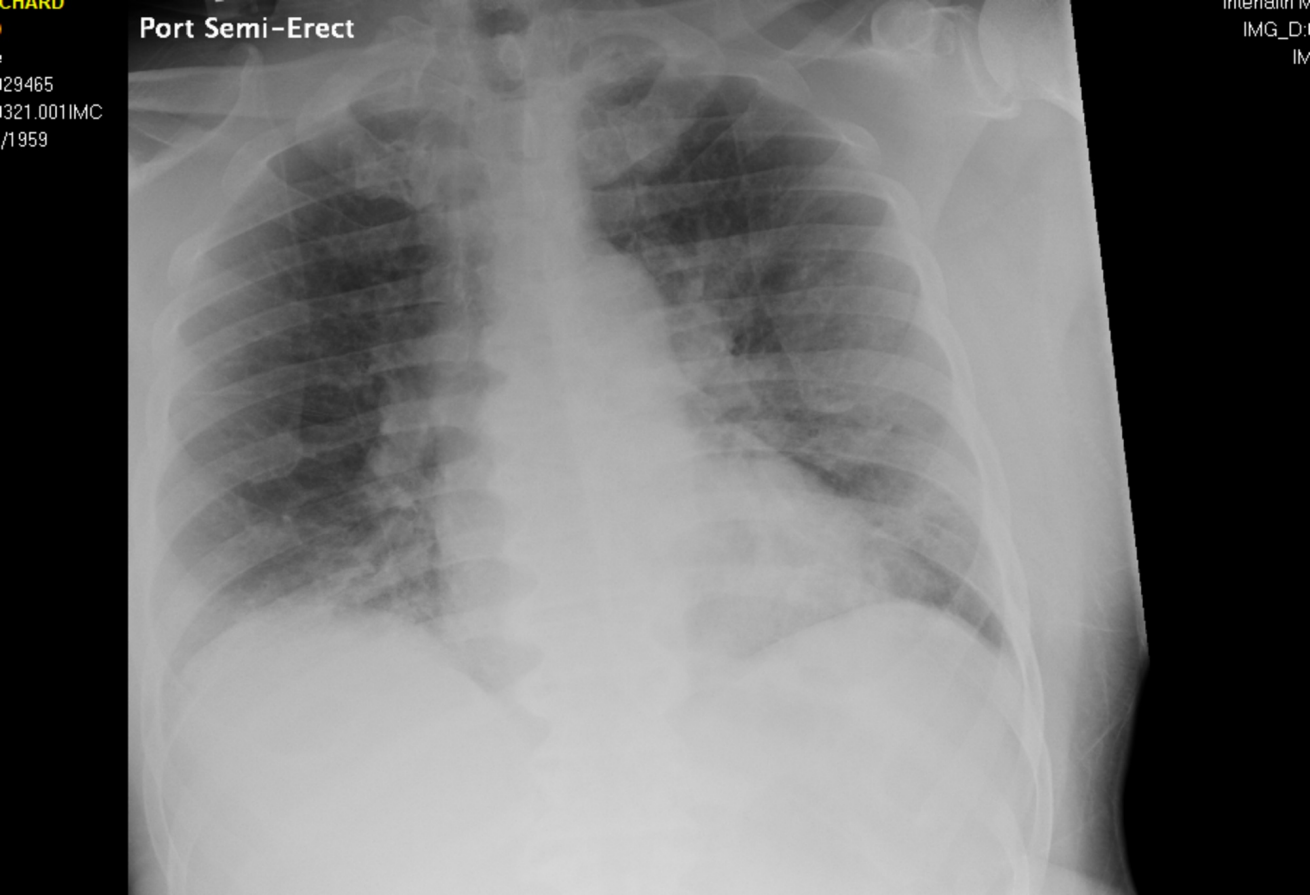
Case 2 chest X-ray on admission April 03, 2020

**Figure 11:**
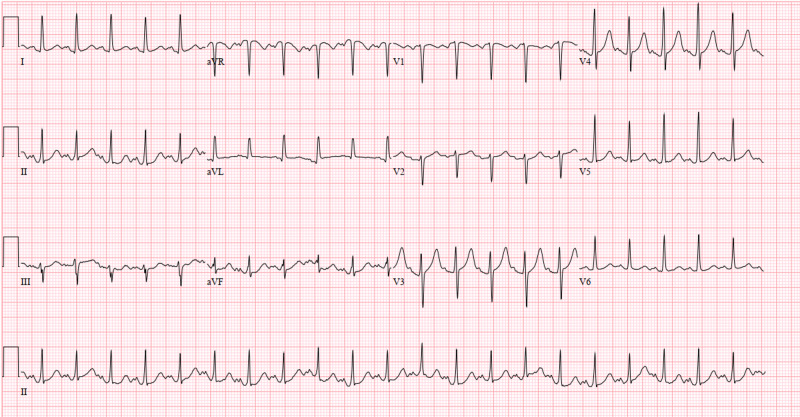
Case 2 electrocardiogram on admission (April 03, 2020)

**Figure 12:**
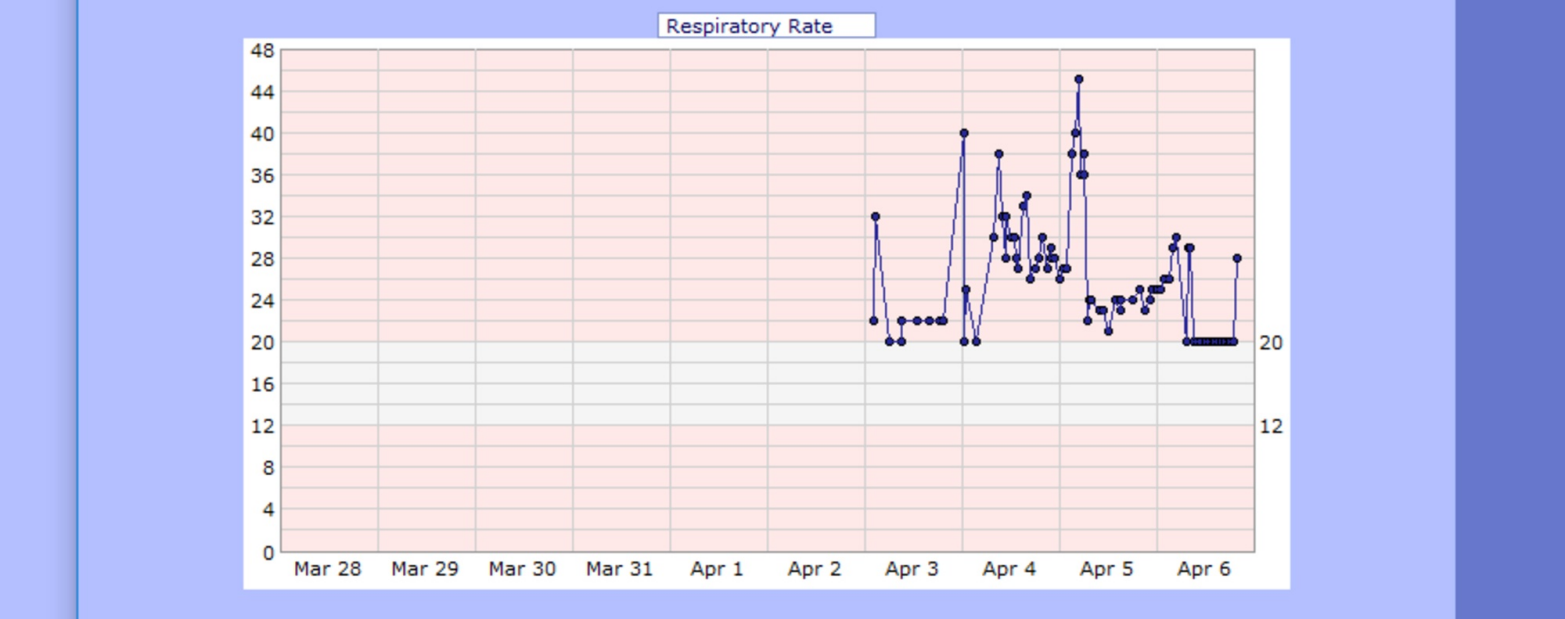
Case 2 respiratory rate from April 03, 2020, to April 06, 2020 (breaths per min)

**Figure 13:**
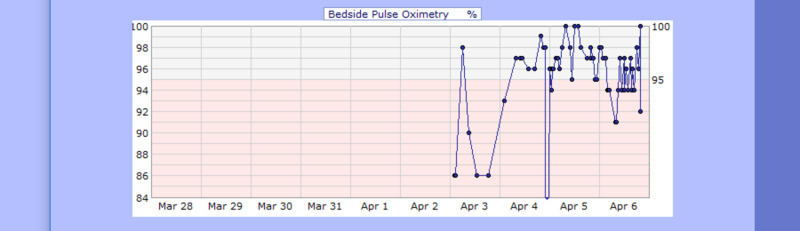
Case 2 pulse oximetry from April 03, 2020, to April 06, 2020 (%)

**Figure 14:**
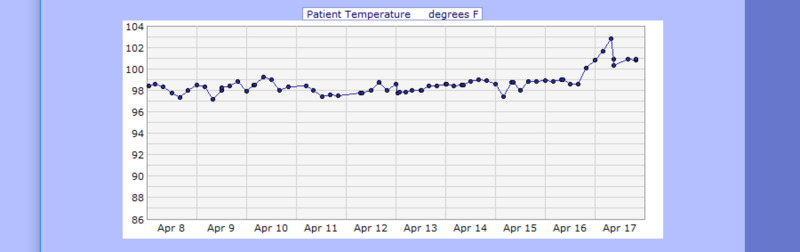
Case 2 temperature from April 08, 2020, to April 17, 2020 (F˚)

On April 03, 2020, a 60-year-old male with a known medical history of hypertension presented to the emergency department (ED) complaining of feeling sick for the past two weeks, having severe, persistent, and non-bloody diarrhea. Additionally, he reported having some fever and chills, persistent dry cough, and worsening shortness of breath for the past few days. His initial chest X-ray was concerning for multilobar pneumonia with commonly reported imaging features of COVID-19 pneumonia (Figure 10), and his electrocardiogram showed sinus tachycardia with a corrected QT interval of 457 ms (Figure 11). His oxygen saturation on room air was 86%; therefore, he was put on a rebreather mask. However, he developed respiratory distress causing his oxygen saturation to drop (Table 3, Figure 12, Figure 13). Consequently, he was intubated and upgraded to the intensive care unit (on pressure-regulated volume control (PRVC) with tidal volume 500 ml, FiO2 of 100%, positive end-expiratory pressure of 5 mmHg (PEEP), and respiratory rate 20/min). Additionally, COVID-19 RT-PCR came back positive, and he was started on the following medications: hydroxychloroquine, azithromycin, (later replaced by doxycycline due to the QT interval), hydrocortisone 100 mg (then replaced by methylprednisone 40 mg), and heparin 5000 units Q12H for deep vein thrombosis (DVT) prophylaxis (later replaced with enoxaparin 30 mg daily on April 9, 2020). He was put on cardiac monitoring in light of elevated troponin (troponin 0.14 ng/mL) and administration of hydroxychloroquine while electrolyte levels were monitored. Other laboratory findings are shown in Table 2. His kidney function deteriorated; therefore, he underwent hemodialysis (HD) on April 7, 2020 (blood urea nitrogen (BUN)/Cr: On admission 41/1.9; Pre-HD 120/9.9; Post-HD 78/6.01). On April 17, 2020, his temperature spiked to 102 F˚ (Figure 14), and his white blood cell (WBC) was elevated; consequently, blood culture (which grew Staphylococcus hominis), transtracheal aspiration (TTA), and urine analysis (UA; which grew Candida albicans) was sent, and he was started on vancomycin and meropenem. These were discontinued on April 21, 2020, and replaced with piperacillin/tazobactam 2.25 gm Q6H and fluconazole due to elevated liver function tests (LFTs) (Table 2). Additionally, his potassium level was elevated to 6.0 mmol/L and glucose was 591 mg/dL; therefore, he was treated with calcium gluconate and insulin. Enoxaparin was held on April 17, 2020, due to the presence of bloody secretion in the nasogastric tube (NGT). Apixaban 2.5 mg twice a day was started on April 19, 2020, after hemoglobin levels remained stable and the bloody secretions ceased.

**Table 3:** Case 2 Arterial blood gas from April 03, 2020, to April 21, 2020 ABG: arterial blood gas; pCO2: partial pressure of carbon dioxide; pO2: partial pressure of oxygen; FiO2: fractional inspired oxygen; P/F: arterial oxygen partial pressure/fractional inspired oxygen

Date	4/3/2020	4/9/2020	4/10/2020	4/21/2020
PH	7.244	7.177	7.143	7.316
pCO2	40.0	50.3	67.4	48.4
pO2	108	79.9	117	84.3
O2 saturation	96.7%	93.5%	96.9%	94.5%
O2 delivery	Ventilator	Ventilator	Ventilator	Ventilator
FiO2	100%	60%	50%	40%
P/F ratio	108	133	234	210

**Figure 15:**
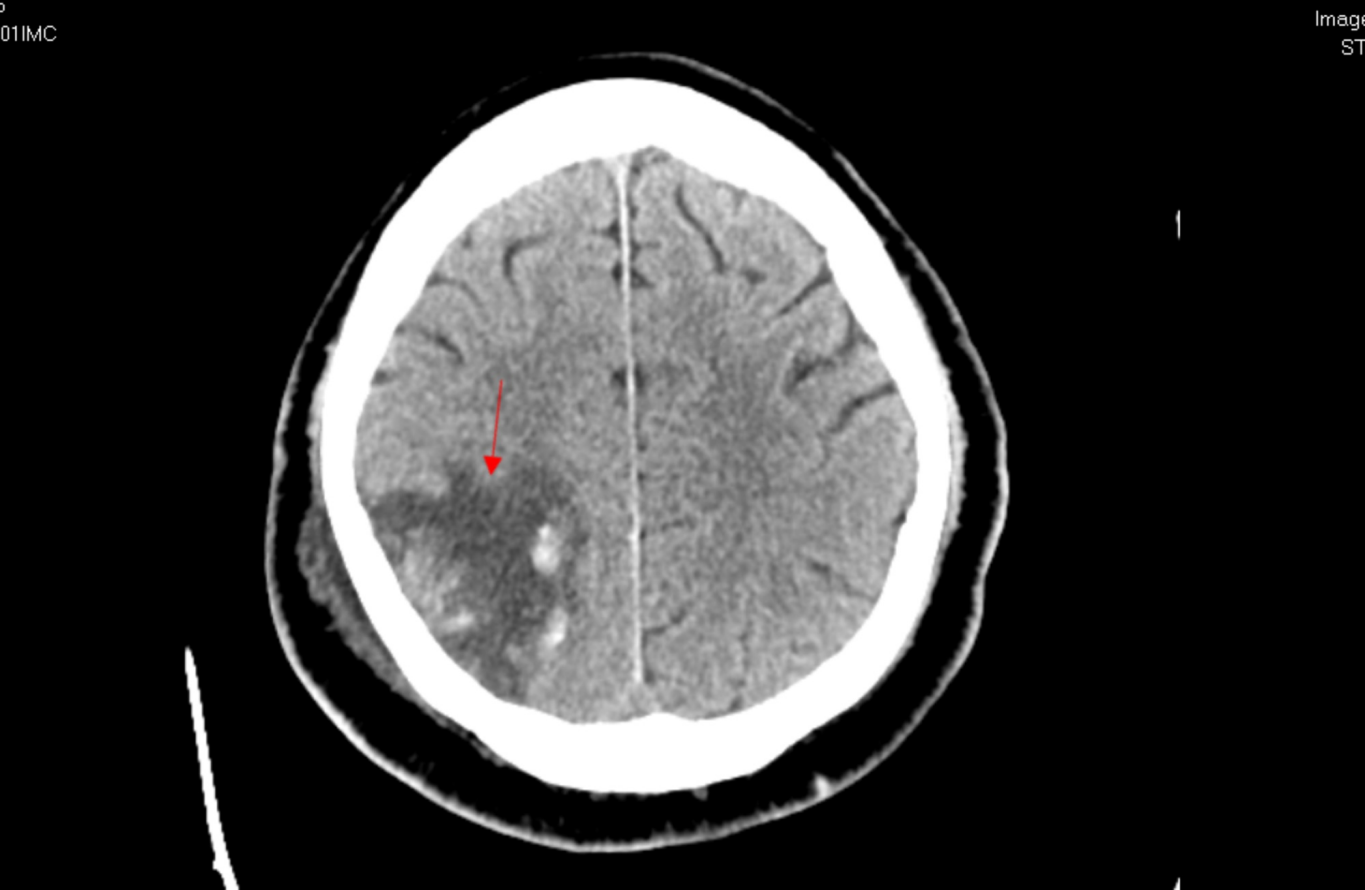
Case 2 head CT on April 22, 2020

On April 22, 2020, he was unresponsive to painful stimuli; therefore, a head CT was ordered, which showed a low-density area in the posterior right cerebral hemisphere with overlying subarachnoid hemorrhages representing a subacute infarction with associated hemorrhages in the absence of head trauma (Figure 15). Neurology consult advised the transfer of patients to a neurosurgery facility for further care.

Case 3

**Figure 16:**
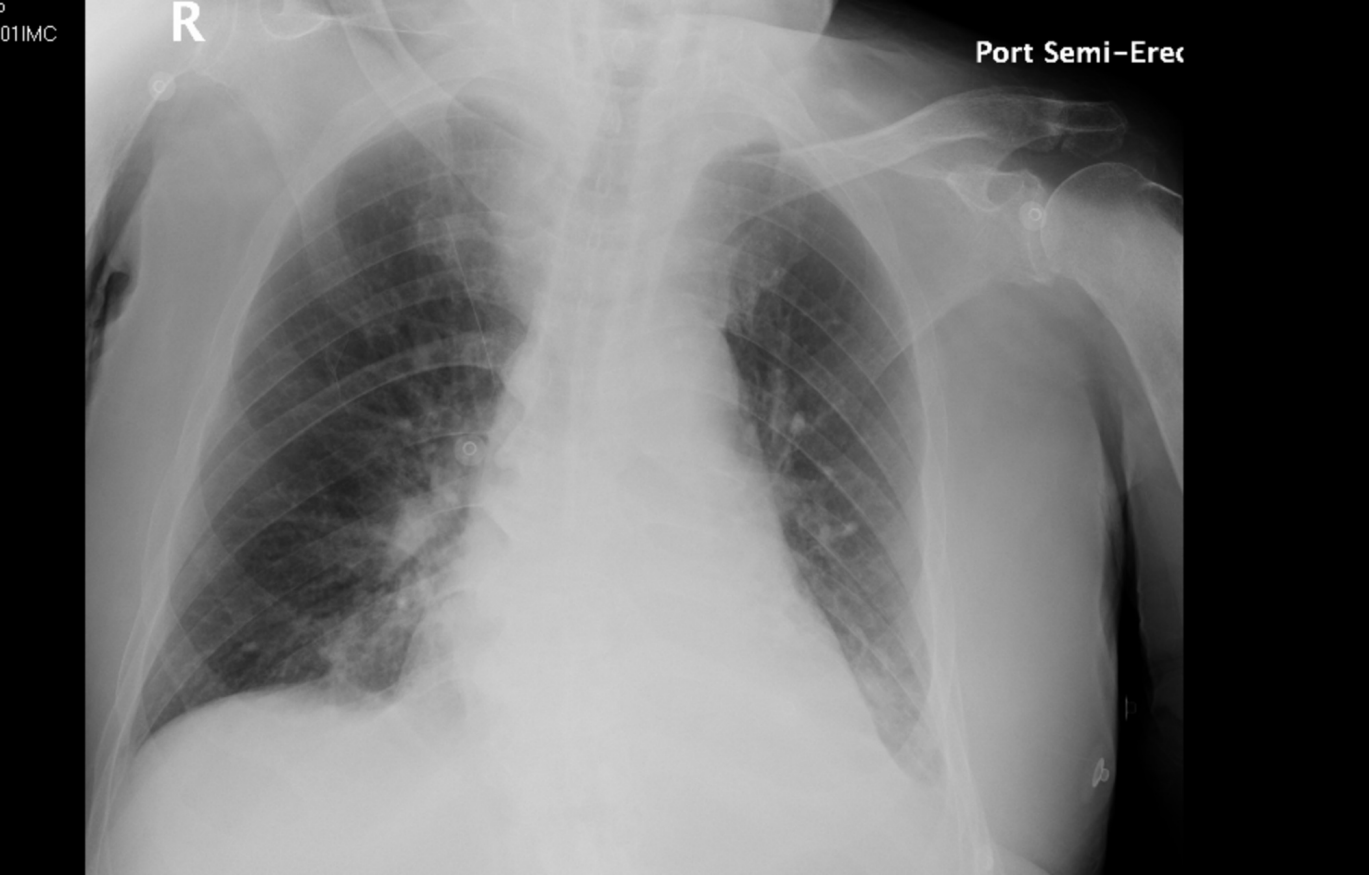
Case 3 chest X-ray on admission on April 12, 2020

**Figure 17:**
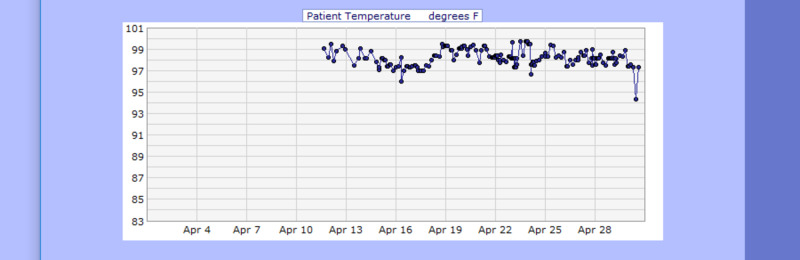
Case 3 temperature from April 12, 2020, to April 30, 2020 (F˚)

**Figure 18:**
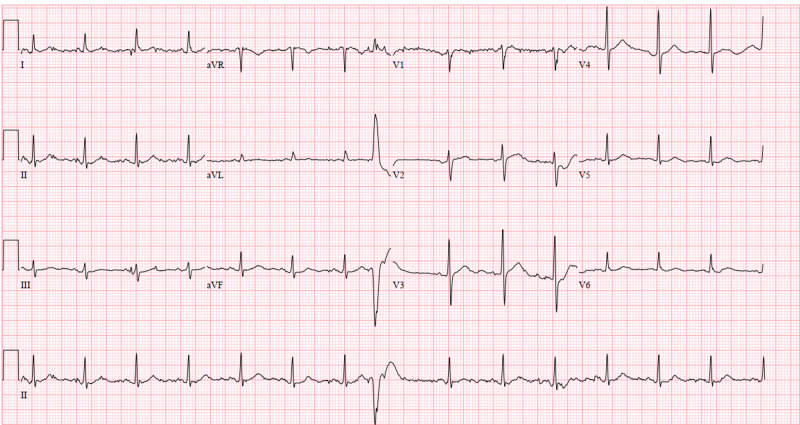
Case 3 electrocardiogram on admission (April 12, 2020)

**Figure 19:**
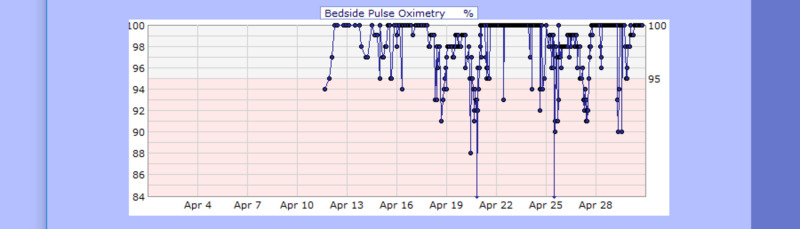
Case 3 pulse oximetry from April 12, 2020, to April 30, 2020 (%)

On April 12, 2020, a 74-year-old African American male with a known medical history of diabetes and hypertension presented to the emergency department by paramedics for dry cough, three days of fatigue, and one day of shortness of breath. The patient denied any fever and night sweats. His chest X-ray showed cardiomegaly with pulmonary vascular congestion, small left pleural effusion, and bibasilar airspace disease. Findings of secondary superimposed pneumonia due to congestive heart failure could not be excluded (Figure 16). Even though he was afebrile (Figure 17), a SARS-CoV-2 RT-PCT test was performed due to the recent outbreak of COVID-19, which came back positive. Furthermore, the electrocardiogram showed sinus rhythm with ventricular premature beats and a corrected QT interval of 449 ms (Figure 18). His troponin was elevated (0.05 ng/mL), brain natriuretic peptide (BNP) was <10 pg/mL, and his O2 saturation was low (94%) (Figure 19). Consequently, he was admitted for cardiac and respiratory monitoring; he was put on a non-rebreather mask (40% oxygen), started on azithromycin (500 mg intravenous (IV) daily), ceftriaxone (1 gm IV daily), furosemide (40 mg IV daily), methylprednisolone (40 mg IV Q12H), and heparin for deep venous thrombosis (DVT) prophylaxis (5000 units Q8H SQ). Other laboratory findings are presented in Table 2.

**Figure 20:**
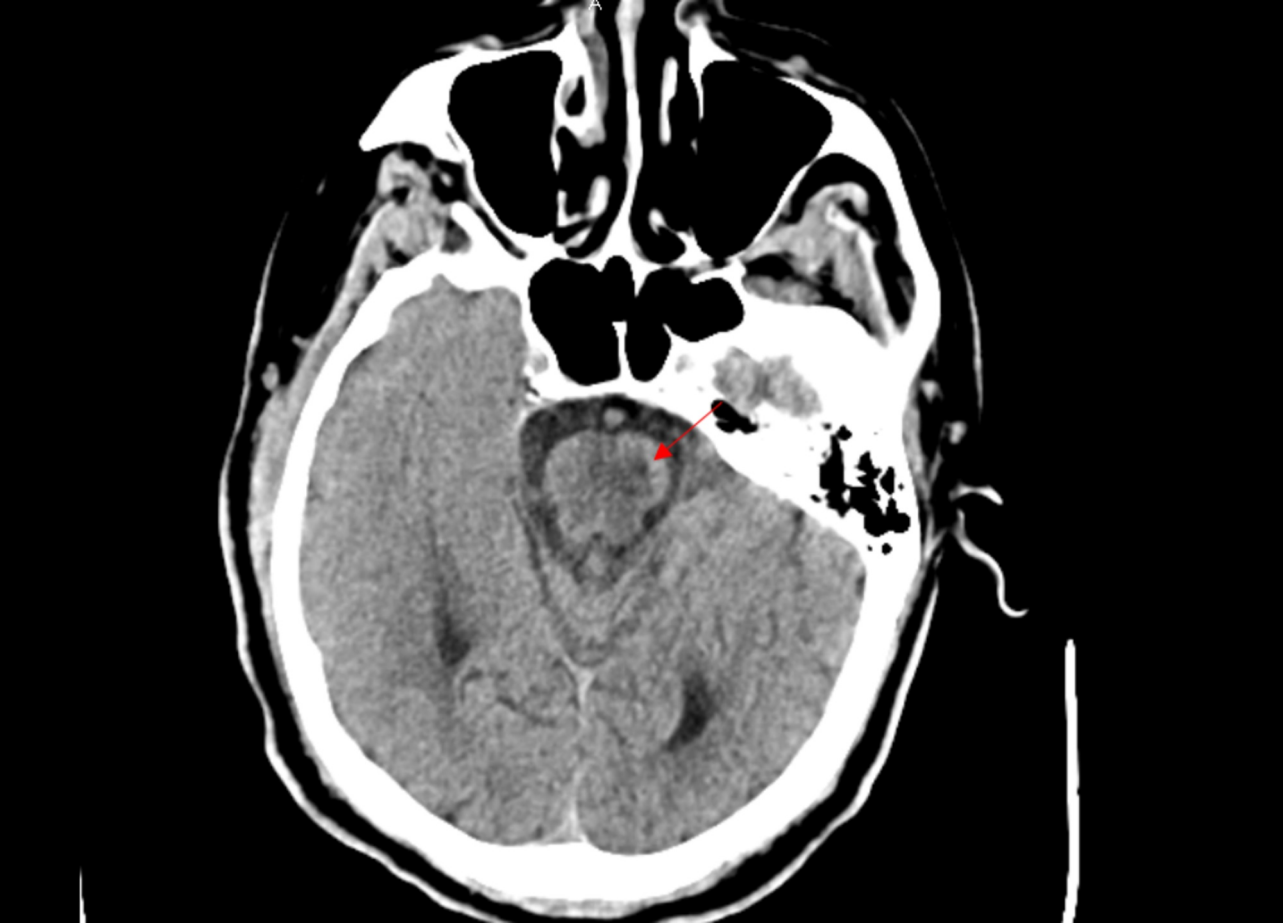
Case 3 head CT on April 25, 2020

On the fourth day of admission, he became hypotensive and was in respiratory distress; his blood pressure dropped to 79/53 mmHg, pulse was 120 bpm, and respiratory rate was 30/min. He was intubated and put on mechanical ventilation (assisted control, tidal volume 400 ml, respiratory rate 15/min, positive end-expiratory pressure (PEEP) 5, and FiO2 50%) and admitted to the ICU (ABG results in Table 4). He also developed AKI (4/18/202 BUN 91.7 mg/dL and Cr 2.73 mg/dL). On day 9, he had poor mentation even after sedation was held. A head CT was then ordered and reported focal area of hypodensity within the pons mainly along the left side, which indicated possible age-indeterminate infarction (Figure 20). Methylprednisolone was held, and heparin was switched to apixaban (5 mg PO BID). His clinical status remained poor and worsened. Subsequently, on April 30, 2020, he went into cardiac arrest and passed away.

**Table 4:** Case 3 Arterial blood gas from April 12, 2020, to April 29, 2020 ABG: arterial blood gas; pCO2: partial pressure of carbon dioxide; pO2: partial pressure of oxygen; FiO2: fractional inspired oxygen; P/F: arterial oxygen partial pressure/fractional inspired oxygen

Date	4/12/2020	4/16/2020	4/17/2020	4/20/2020	4/29/2020
PH	7.411	7.382	7.398	7.285	7.420
pCO2	42	46.5	39.1	58.6	38.2
pO2	201	203	129	49.7	78.3
O2 saturation	99%	99.2%	98.5%	81.8%	95.8%
O2 delivery	V.mask	Ventilator	Ventilator	Ventilator	Ventilator
FiO2	40%	100%	40%	100%	40%
P/F ratio	502	203	322	49.7	195.7

## Discussion

The pathophysiology of severe acute respiratory syndrome due to coronavirus 2 (SARS-CoV-2), is the overproduction of early response proinflammatory cytokines, primarily tumor necrosis factor (TNF), interleukin-6, and interleukin-1β. The result is what has been described as a cytokine storm, leading to an increased risk of vascular hyperpermeability, multiorgan failure, and, in some cases, death [2-3]. Laboratory studies have confirmed “respiratory infections as triggers for acute myocardial infarction and stroke.” Data have already shown strokes triggered by respiratory infections such as the influenza virus and other confirmed respiratory viruses [4].

The high concentrations of proinflammatory cytokines in severe COVID-19 cases induce endothelial and mononuclear cell activation with the expression of tissue factor, ultimately causing the activation of coagulation and the production of thrombin. Moreover, high levels of thrombosis and inflammatory serum markers, such as D-dimer, fibrinogen, and CRP, have been seen in COVID-19 patients. During the inflammatory process, concentrations of antithrombin III, tissue factor pathway inhibitor, and the protein C system may decline, causing an imbalance in the anticoagulation system. The combination of these factors induces a pro-coagulation state and predisposes patients to microthrombosis and venous and arterial thromboembolism [2-3,5].

Furthermore, in a case series by Kochi et al., five of six COVID-19 patients with ischemic stroke showed the production of antiphospholipid antibodies (aPL) [5]. Other case reports have also demonstrated evidence of coagulopathy and antiphospholipid antibodies in COVID-19 patients who developed ischemic strokes. These findings suggest an additional avenue in which COVID-19 can induce a hypercoagulable state and, consequently, ischemic stroke [5-6].

Multiple other reports have shown incidences of stroke in COVID-19 patients. A retrospective study of data from the COVID-19 outbreak in Wuhan, China, revealed an approximately 5% incidence of stroke in hospitalized patients with COVID-19 [7]. Other reports suggest a higher rate of cerebrovascular disease (mainly ischemic stroke) in severe COVID-19 patients as compared to non-severe cases [5]. Additionally, case studies have shown large-vessel occlusion, with some in multiple territories [5]. A case series by Warren-Gash et al. reported cases of COVID-19 positive patients with large-vessel strokes in five patients younger than 50 years of age [8].​​​​​​

## Conclusions

We propose that COVID-19 infection influences the coagulation system, therefore, increasing the risk of stroke in patients, especially those with other risk factors for stroke such as hypertension and diabetes. Additionally, we propose that careful monitoring and administration of anticoagulant therapies is necessary. Further studies and trials are required to confirm these hypotheses.
